# Vertical Distribution of Ozone and Nitric Acid Vapor on the Mammoth Mountain, Eastern Sierra Nevada, California

**DOI:** 10.1100/tsw.2002.72

**Published:** 2002-01-05

**Authors:** Andrzej Bytnerowicz, David R. Parker, Pamela E. Padgett

**Affiliations:** USDA Forest Service, Pacific Southwest Research Station, 4955 Canyon Crest Drive, Riverside, CA 92507, USA; Department of Environmental Sciences, University of California, Riverside, CA 92521, USA

## Abstract

In August and September 1999 and 2000, concentrations of ozone (O_3_) and nitric acid vapor (HNO_3_) were monitored at an elevation gradient (2184–3325 m) on the Mammoth Mountain, eastern Sierra Nevada, California. Passive samplers were used for monitoring exposure to tropospheric O_3_ and HNO_3_ vapor. The 2-week average O_3_ concentrations ranged between 45 and 72 ppb, while HNO_3_ concentrations ranged between 0.06 and 0.52 μg/m3. Similar ranges of O_3_ and HNO_3_ were determined for 2 years of the study. No clear effects of elevation on concentrations of the two pollutants were detected. Concentrations of HNO_3_ were low and at the background levels expected for the eastern Sierra Nevada, while the measured concentrations of O_3_ were elevated. High concentrations of ozone in the study area were confirmed with an active UV absorption O_3_ monitor placed at the Mammoth Mountain Peak (September 5–14, 2000, average 24-h concentration of 56 ppb).

## INTRODUCTION

Ambient tropospheric ozone ($O_3$) results from production in the free troposphere, injection from the stratosphere to the troposphere, photochemical production, photolysis, dry deposition, and chemical destruction[1]. Stomatal uptake of vegetation, especially by forest canopies, may significantly affect ambient $O_3$ concentrations[2]. First measurements of tropospheric $O_3$ concentrations made between 1876 and 1910 at Montsouris near Paris showed background levels of ~10 ppb[3]. Concentrations of $O_3$ increase gradually in the Northern Hemisphere — over the last 30 years, 1–2% annual increase of $O_3$ concentrations has been recorded in Europe[4]. At present, $O_3$ concentrations of >50–60 ppb are often measured as the background levels[5]. $O_3$ is a criteria pollutant affecting human health at elevated concentrations[6]. It has been well established that $O_3$ may be toxic to vegetation at concentrations >30–40 ppb and that severity of plant damage depends on a length and characteristics of the exposure and various abiotic and biotic factors[7,8,9].

In dense urban areas such as southern California, nitrogen oxides are a major component of photochemical smog. During the photochemical smog formation process, nitric oxide (NO) is rapidly oxidized to nitrogen dioxide ($NO_2$) that subsequently reacts with hydroxyl radicals producing nitric acid ($HNO_3$) vapor[10,11]. While $NO_2$ is often the dominant nitrogenous (N) pollutant[12,13], $HNO_3$ vapor may be more relevant to problems associated with N deposition to forests and other ecosystems because of its unusually high deposition velocity[14,15]. In areas of high ambient concentrations, $HNO_3$ may also exhibit direct toxic effects on foliage of forest vegetation[16].

Concentrations of $O_3$ and $HNO_3$ vapor in areas affected by photochemical smog are strongly correlated in several locations in the Los Angeles Basin[17]. However, in mountain locations down-wind from the photochemical source areas, concentrations of $HNO_3$ decrease more rapidly than those of $O_3$ due to high deposition velocity of $HNO_3$ vapor[18].

There is a rapidly growing interest in passive sampling systems for quantifying exposure to ambient concentrations of gaseous air pollutants. Excluding the laboratory analysis costs, passive samplers are inexpensive, easy to use, and do not require electricity to operate. Therefore, they are very attractive for use in remote and wilderness areas and for regional scale air-quality assessments[19]. Application of passive samplers has allowed acquisition of important information on spatial and temporal distribution of $O_3$ exposure for the entire Sierra Nevada range[20,21] and $O_3$, $NH_3$, $NO_2$, and $HNO_3$ in Sequoia National Park[22].

Sierra Nevada Mountains are the primary topographic feature of the state of California. These mountains have tremendous ecological, economical, and recreational values[23]. Sierra Nevada is quite diversified in regard to air pollution distribution. Western slopes of the Sierra Nevada are strongly affected by pollution generated in the San Francisco Bay and Central Valley[24], while the eastern Sierra Nevada has been regarded as a clean area with relatively low pollutant concentrations[25,26,27]. The Mammoth Mountain near Mammoth Lakes in Eastern Sierra Nevada (Fig. 1) is a popular recreational location for millions of Californians, as well as visitors from other states and abroad.

## METHODOLOGY

Air pollution monitoring on the Mammoth Mountain was performed during the summer class "Acquisition and Analysis of Environmental Data," organized by the Department of Environmental Sciences of the University of California in Riverside. The $O_3$ passive samplers[28] produced by Ogawa & Co., USA, Inc., and $HNO_3$ samplers[30] were used. The samplers were exposed from August 20 to September 7, 1999, and from August 21 to September 6, 2000, at locations presented in Fig. 2 and described in Table 1. Passive samplers were placed about 2 m above the ground on PVC poles — each sampler contained two replicate filters subsequently used for analyses. Nitrate from the $O_3$ samplers' filters, a product of nitrite oxidation by $O_3$, was extracted with nanopure water and determined with ion chromatography (Dionex 4000i)[28,29]. Nitrate from nylon filters, a product of $HNO_3$ absorption, was also extracted with nanopure water, and its concentrations were determined colorimetrically (TRAACS 2000 Bran & Lueble Instrument)[30,31]. $O_3$ passive samplers were calibrated against a UV-absorption instrument (Thermo Environmental Model 49) located nearby in Yosemite National Park. $HNO_3$ samplers were calibrated against honeycomb denuder systems in continuously stirred tank reactor (CSTR) chambers located at the University of California in Riverside[32]. Between September 5 and 14, 2000, concentration of $O_3$ at the Mammoth Mountain Peak were also measured with a 2B Technologies UV-absorption instrument[33].

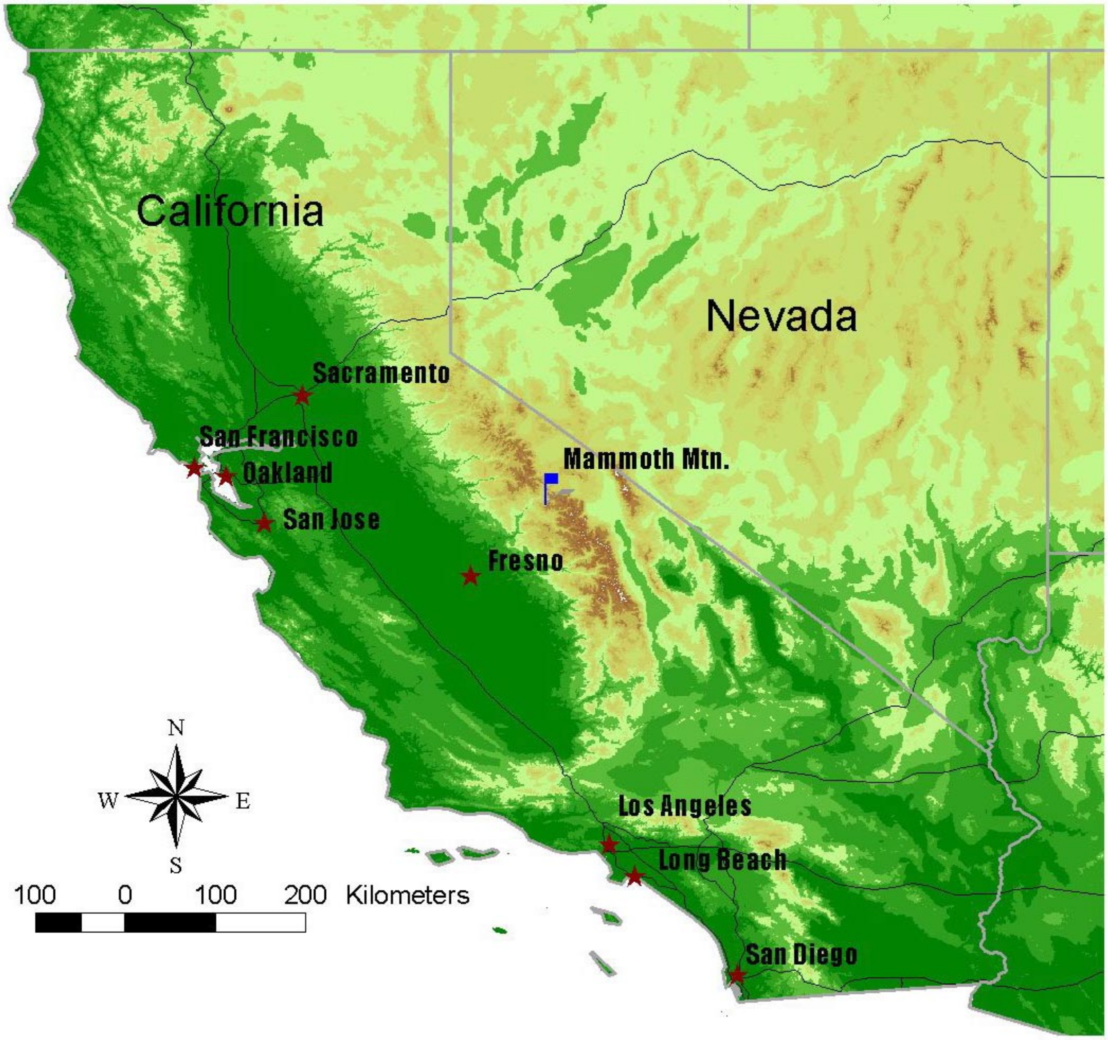

**FIGURE 1**. Location of Mammoth Mountain in Sierra Nevada, California.

**TABLE 1**
**Location and Elevation of Monitoring Sites on Mammoth Mountain**

| Site Number | Name | Elevation (m) | |
|---|---|---|---|
| | | 1999 | 2000 |
| 1 | Rainbow Falls | 2267 | 2184 |
| 2 | Starkweather Lake | 2416 | 2416 |
| 3 | Agnew Meadows | 2591 | 2688 |
| 4 | Minaret Summit | 2810 | 2810 |
| 5 | Chair 14 | 3152 | 3152 |
| 5B | Deadman’s Pass | - | 3135 |
| 6 | Mammoth Mountain Summit | 3350 | 3325 |

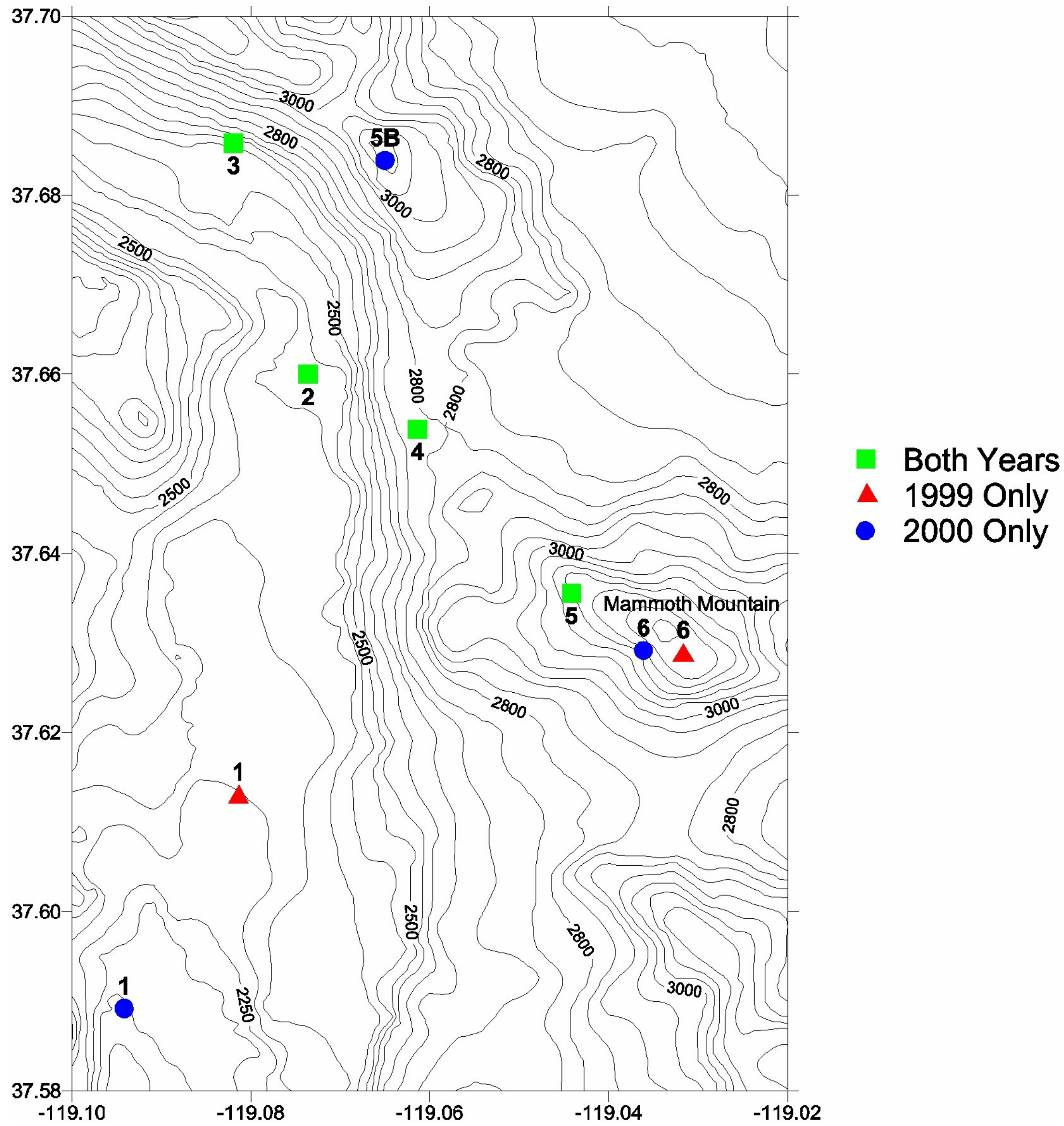

**FIGURE 2**. Location of monitoring sites on Mammoth Mountain.

## RESULTS AND DISCUSSION

### Ozone

Concentrations of $O_3$ in 1999 increased with elevation up to 2810 m (Minaret Summit site), decreased at 3152 m (Chair 14), and increased to the highest level of 72 ppb at 3330 m (Mammoth Mountain Peak). In 2000 no clear effect of elevation on $O_3$ concentrations was recorded. $O_3$ concentrations at the 2416-, 2688-, 2810-, and 3152-m sites (Starkweather Lake, Agnew Meadows, Minaret Summit and Chair 14, respectively) were similar to the 1999 values. However, when in 2000 the Rainbow Falls site was moved to the more exposed but lower elevation location, the $O_3$ concentrations significantly increased. On the other hand, when location of the passive sampler on the Mammoth Mountain Peak was moved 25 m lower, the $O_3$ concentrations decreased (Fig. 3).

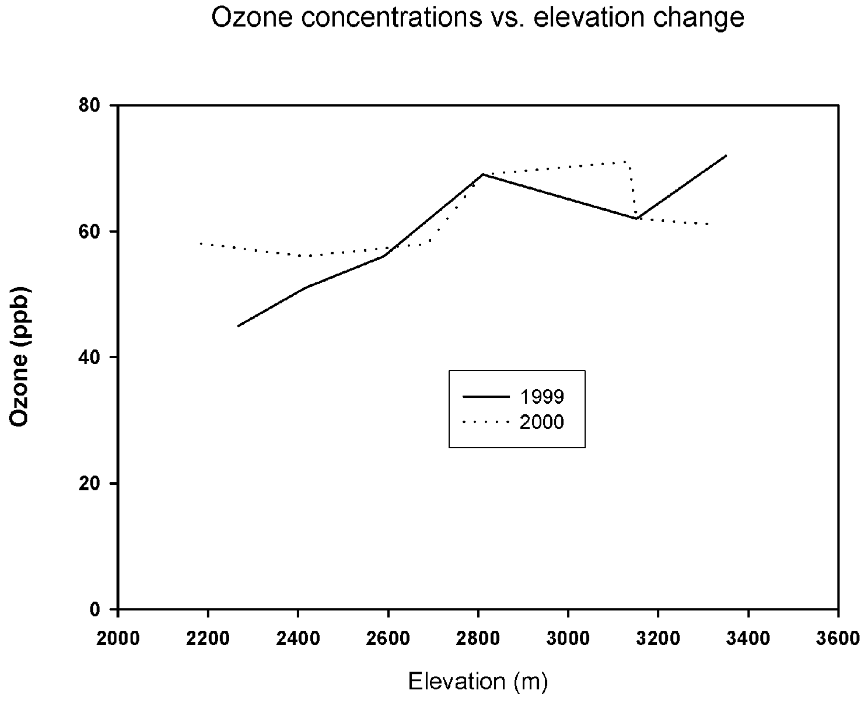

**FIGURE 3**. Concentrations of $O_3$ determined with passive samplers on Mammoth Mountain.

Measurements of $O_3$ concentrations with an active monitor at the Mammoth Mountain Peak confirmed high levels of the pollutant determined with passive samplers near that site during 1999 and 2000 measurements. Diurnal distribution of $O_3$ concentrations varied between individual days without any particular pattern. Examples of diurnal distribution for 4 days are presented on Fig. 4. The lowest recorded value was 35 ppb on September 12, while during other days $O_3$ levels stayed >40 ppb. On September 8 the $O_3$ levels reached 73 ppb. $O_3$ average concentration for the entire period of active monitoring was 56 ppb (S.D. 10 ppb). These values are similar to those recorded in August 1987 at the nearby Eastern Brook Lake in the eastern Sierra Nevada[34]. These concentrations can be considered as elevated above normal background concentrations for the Sierra Nevada — in other parts of the Sierra Nevada range, far from the photochemical smog source areas, 2-week average $O_3$ concentrations stayed ~40 ppb[35,36]. Persistence of high $O_3$ levels and a lack of clear diurnal patterns indicated that the pollutant originated in a remote pollution source area, either in California Central Valley or southern California. Transport of polluted air masses from southern California through the Tehachapi and Cajon Passes takes place[37], therefore increased levels of the pollutant along the eastern Sierra Nevada should be considered. If these were true, the expected concentrations of $O_3$ at sites located more to the south and closer to the source area would be higher. However, results of recent air-quality measurements indicate that concentrations of $O_3$ in the Bishop area (maximum 8-h average concentrations ~60 ppb) are much lower than at Mammoth Lakes located about 70 km to the north (maximum 8-h average concentrations ~100 ppb)[38]. Therefore, a possibility of transport of the polluted air masses from California Central Valley across the Sierra Nevada seems to be more plausible. We suggest that polluted air masses from the Fresno area in the San Joaquin Valley (maximum 8-h $O_3$ average concentrations approaching 126 ppb) may be transported with prevailing summer winds[39] along the San Joaquin River drainage into the Mammoth Mountain area).

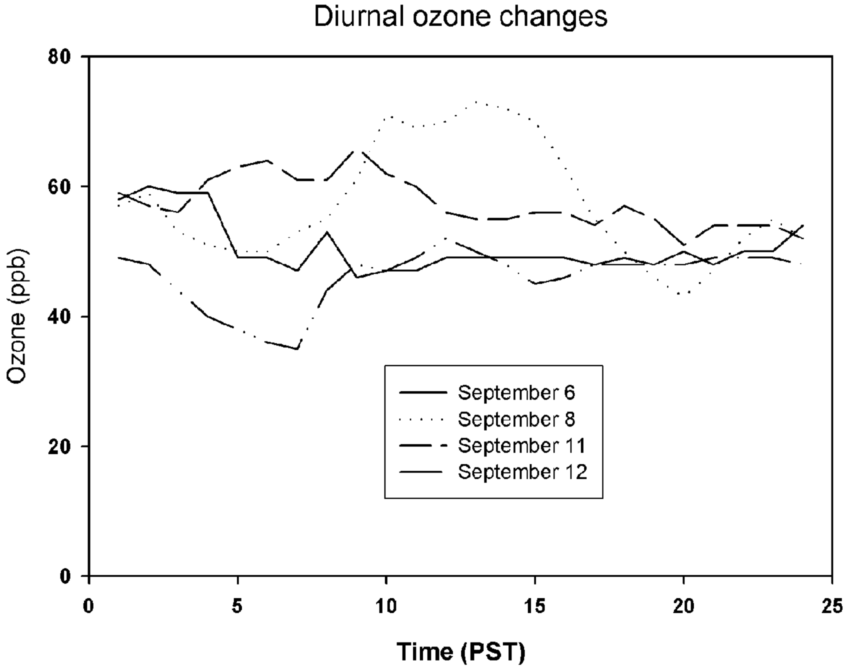

**FIGURE 4**. Concentrations of $O_3$ determined with an active UV absorption monitor at the Mammoth Mountain Peak.

## Nitric Acid Vapor

During the 1999 study $HNO_3$ concentrations increased with elevation with an exception of Agnew Meadows (2591 m) that was as low as the lowest-elevation Rainbow Falls site (2267 m). In 1999 the $HNO_3$ concentrations ranged between 0.06 and 0.52 $\mu g/m^3$. During the 2000 study no clear relationship with elevation was detected, but ranges of concentrations remained within those seen in 1999 (0.25–0.42 $\mu g/m^3$). In general, the detected $HNO_3$ concentrations were at low background levels and similar to 0.36 $\mu g/m^3$ summer average detected in the nearby Eastern Brook Lakes[27]. These results are also similar to other remote mountain locations in North America[40]. In the western Sierra Nevada $HNO_3$ levels are higher — at Whitaker Forest the 24-h average daytime $HNO_3$ concentration in summer of 1990 was ~1.1 $\mu g\ m^{-3}$[41]. At high elevation locations in Sequoia National Park, 2-week long average $HNO_3$ concentrations ranged between 0.04 and 1.4 $\mu g/m^3$ in summer 1999[42]. In the moderately polluted Barton Flat site of the San Bernardino Mountains, the average 24-h concentrations $HNO_3$ ranged from 3.0 to 6.5 $\mu g\ m^{-3}$ during the 1993–1995 summer seasons[16]. Although we assume that polluted air from the San Joaquin Valley contained high concentrations of $O_3$, concentrations of $HNO_3$ were drastically depleted compared to the source area due to a very high reactivity of the pollutant[14] and deposition to rocks and vegetation before reaching the receptor area. At the $HNO_3$ levels recorded at the Mammoth Mountain, no phytotoxic effects or significant levels of nitrogen deposition to natural ecosystems can be expected[43].

This study indicated that application of relatively simple techniques such as passive sampling offers new opportunities for evaluation of air quality in remote locations. Such information is urgently needed, especially for land managers of ecologically important areas. The Mammoth Mountain is in close vicinity of the John Muir and Ansel Adams Wilderness areas. These important Class I areas require special federal protection and should have good air quality for protection of sensitive flora and fauna and for well-being of numerous local inhabitants and visitors coming for recreation and rest.

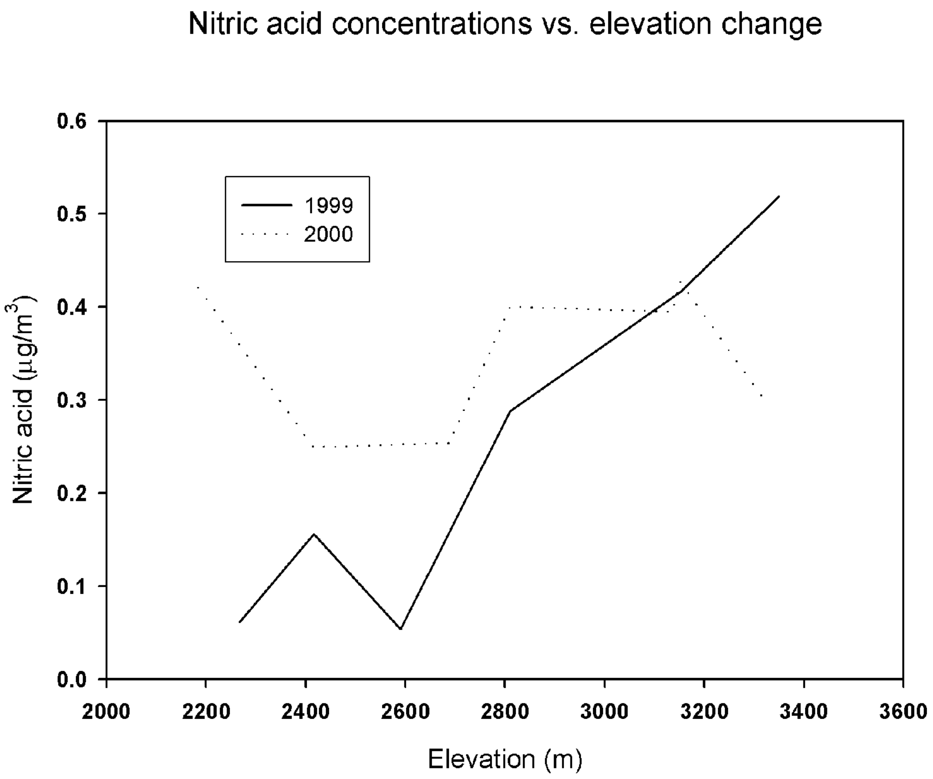

**FIGURE 5**. Concentrations of $HNO_3$ determined with passive samplers on the Mammoth Mountain.

## CONCLUSION

The Mammoth Mountain in the eastern Sierra Nevada is exposed to $O_3$ concentrations that are elevated above background. This was not expected in this location, which is distant and separated from the photochemical smog source areas by the Sierra Nevada range. A possibility of a trans–Sierra Nevada transport of polluted air masses from the polluted San Joaquin Valley is high.

$HNO_3$ vapor concentrations, however, were low and at the expected background levels may not pose a threat to natural resources or to humans of the investigated area.

This study showed that passive samplers are very useful for monitoring of air pollutants in remote mountain locations. Such samplers can also serve as simple educational tools for students interested in environmental pollution problems.

## ACKNOWLEDGMENTS

We thank students of the UC Riverside ES 176 class for participation in data collection. We also thank Ed Betty of the University of California in Riverside, Environmental Sciences Department, and Diane Alexander of the USDA Forest Service Pacific Southwest Research Station in Riverside, CA, for chemical analyses of the collected samples, as well as Susan Schilling of the USDA Forest Service Pacific Southwest Research Station for help in preparation of graphics. Fig. 1 was produced based on data distributed by EROS Data Center Distributed Active Archive Center (EDC DAAC), located at the U.S. Geological Survey's EROS Data Center in Sioux Falls, SD.
